# Sol–Gel Synthesis of Silica–Poly (Vinylpyrrolidone) Hybrids with Prooxidant Activity and Antibacterial Properties

**DOI:** 10.3390/molecules29112675

**Published:** 2024-06-05

**Authors:** Albena Bachvarova-Nedelcheva, Yoanna Kostova, Lilia Yordanova, Elena Nenova, Pavletta Shestakova, Iliana Ivanova, Elitsa Pavlova

**Affiliations:** 1 Institute of General and Inorganic Chemistry, Bulgarian Academy of Sciences, Acad. G. Bonchev Str., Bl. 11, 1113 Sofia, Bulgaria; 2 Institute of Metal Science, Equipment and Technologies with Hydro- and Aerodynamics Centre “Acad. A. Balevski”, Bulgarian Academy of Sciences, Shipchenski Prohod Str., 67, 1574 Sofia, Bulgaria; y_kostova@ims.bas.bg; 3 Faculty of Biology, Sofia University “St. Kliment Ohridski”, 8 Dragan Tsankov Blvd., 1164 Sofia, Bulgaria; lilijapj@uni-sofia.bg (L.Y.); nenova@uni-sofia.bg (E.N.); iaivanova@biofac.uni-sofia.bg (I.I.); 4 Institute of Organic Chemistry with Centre of Phytochemistry, Bulgarian Academy of Sciences, Acad. G. Bonchev Str., Bl. 9, 1113 Sofia, Bulgaria; pavletta.shestakova@orgchm.bas.bg; 5 Faculty of Physics, Sofia University “St. Kliment Ohridski”, 5 James Boucher Blvd., 1164 Sofia, Bulgaria; elli_pavlova@abv.bg

**Keywords:** sol–gel, silica hybrids, antibacterial properties, ROS, prooxidant activity, *Daphnia magna*

## Abstract

The present work deals with the sol–gel synthesis of silica–poly (vinylpyrrolidone) hybrid materials. The nanohybrids (Si-PVP) have been prepared using an acidic catalyst at ambient temperature. Tetramethyl ortosilane (TMOS) was used as a silica precursor. Poly (vinylpyrrolidone) (PVP) was introduced into the reaction mixture as a solution in ethanol with a concentration of 20%. The XRD established that the as-prepared material is amorphous. The IR and ^29^Si MAS NMR spectra proved the formation of a polymerized silica network as well as the hydrogen bonding interactions between the silica matrix and OH hydrogens of the silanol groups. The TEM showed spherical particle formation along with increased agglomeration tendency. The efficacy of SiO_2_/PVP nanoparticles as a potential antimicrobial agent against a wide range of bacteria was evaluated as bacteriostatic, using agar diffusion and spot tests. Combined effects of hybrid nanomaterial and antibiotics could significantly reduce the bactericidal concentrations of both the antibiotic and the particles, and they could also eliminate the antibiotic resistance of the pathogen. The registered prooxidant activity of the newly synthesized material was confirmative and explicatory for the antibacterial properties of the tested substance and its synergetic combination with antibiotics. The effect of new hybrid material on Crustacea *Daphnia magna* was also estimated as harmless under concentration of 0.1 mg/mL.

## 1. Introduction

A wide range of materials, such as thin films, particles, or aerogels with numerous possible uses, can be produced using sol–gel chemistry [1]. Nowadays, this process is still being developed and it is used in many different industries to produce sophisticated materials, biomaterials, sensors, smart coatings, and optical devices [2]. The synthesis of organic–inorganic hybrids has been facilitated by the sol–gel method over the past three decades. Its key advantages over alternative methods include low processing temperature and pressure, adaptability, and flexibility [3]. It has been shown that the properties of the hybrid materials include a synergy at the nanometer level with the properties of the starting materials, making these materials appropriate for future technological applications [4]. For this reason, they are usually considered to be innovative advanced materials, with promising applications in many fields, such as optics, biology, mechanics, sensors, electronics, and coatings [5]. Hybrid properties strongly depend on the nature and strength of the interactions between the organic and inorganic components [6]. The bioactivity of sol–gel hybrids was found to be higher than that of materials obtained by other methods [7]. The presence of –OH groups on their surface is responsible for their bioactivities.

A large group of sol–gel materials is based on silicon alkoxides, e.g., tetramethoxysilane (Si(OCH_3_)_4_) or tetraethoxysilane (Si(OC_2_H_5_)_4_). Applying appropriate conditions such as precursor/solvent ratio, precursor/water ratio, and catalyst (pH) leads to the formation of a gel composed of a silica network [8,9,10]. These gels are highly desirable for a variety of applications due to their low density, low thermal conductivity, low dielectric constant, and high specific area [11,12,13]. It has to be noted that native silica aerogels are very brittle, which limits their applications. However, several methods used to strengthen their structure have already been developed, and others are still in the works [14]. These include the gels’ aging process, the use of silica precursors to increase the network’s flexibility, and the addition of polymers to silica aerogel networks [1]. Applying the sol–gel processes, silicon alkoxides are also used for the synthesis of hybrid materials which are organic–inorganic ones. The creation of hybrids, which combine the advantageous aspects of inorganic and organic materials, was aided by developments in materials science. The inorganic phase in the hybrids can change the mechanical and thermal properties of pure organic materials and vice versa. Additionally, hybrid materials exhibit new functionalities such as magnetic and electric properties, increased adsorption, or better structural properties [15,16]. In the past, researchers proposed that the characteristics of the resulting hybrid material are significantly influenced by the ratio of the applied polymer, metal alcoholate, water, and catalysts [17]. The silica-based sol–gel functional products are generally based on polymers. Polyvinylpyrrolidone (PVP) is a macromolecular compound which is often used for the sol–gel production of silica hybrids and the first primary results have been reported by several authors [18,19]. Among polymers, PVP is a preferable compound due to its solubility in water and organic solutions, low toxicity, and high physiological compatibility. Its excellent qualities make it widespread, especially in the fields of medicine, foods, cosmetics, and other areas relevant to human health [20].

The synthesis of alternative materials is a highly favored strategy due to the outbreak of infectious diseases and antibiotic resistance [21,22]. Since the discovery of penicillin, β-lactam antibiotics have been widely used as antibacterial agents because of their broad spectrum of activity and low toxicity. However, the overuse of antibiotics has led to bacteria adapting by developing resistance. Due to this fact, pharmaceutical companies and researchers have devoted efforts to find compounds that do not cause such resistance in bacteria. The strategies adopted so far are not particularly effective and new alternatives are being explored to prevent bacterial resistance [23,24]. And this is where nanomaterials come in. They are used as antimicrobial agents due to their unique physical and chemical properties [25]. Considering the growing problem of bacterial resistance to antibiotics, nanoparticle-based antimicrobial agents are expected to provide alternatives to conventional antibiotics and biocidal chemicals. Due to their toxic properties, metal-containing nanocomposites are good candidates for new antimicrobial products for consumers and patient care [26]. A great potential in nanomedicine has been discovered in the past decade due to the effectiveness of various nanoconjugates against pathogenic microbes. One approach to counter bacterial drug resistance is the application of metal composites, especially at the nanoscale, to control bacterial infections [27]. Several enzymes and mutations in genetic sequences can impede multidrug resistance mechanisms by altering drug efflux from cells, thereby reducing bacterial vulnerability to antibacterial agents [28,29]. Therefore, scientists are developing new ways to control resistant pathogens. Advances in nanotechnology have prompted microbiologists to apply metal nanoparticles as an effective way to control certain pathogenic microbes involved in infectious diseases [30,31]. The combination of antibiotics and metal nanoparticles can enhance the efficacy of antibiotics against resistant pathogens. Furthermore, nanoparticle–antibiotic conjugates reduce the amount of both agents in the dose, which reduces toxicity and increases antimicrobial properties [32,33]. These conjugates can be very effective against resistant bacteria. Furthermore, due to this conjugation, antibiotic concentrations are decreased at the site of antibiotic–bacteria contact, and thus the association between microorganisms and antibiotics is accelerated [22,33,34].

The generation of reactive oxygen species (ROS) is an important biomarker about the redox properties of substances. By following the kinetics of the generation of free radicals and ROS, the properties of newly synthesized materials and their effects on the cascade of reactions causing the formation and accumulation of antibacterial free radicals and ROS can be evaluated [35,36]. Chemiluminescence analysis is a rapid and sensitive method for such studies. It is applied to follow the dynamics of free radical and ROS generation and to determine the prooxidant/antioxidant activity of various materials. The responses are recorded in the range of 480–580 nm and can be used to estimate the quantum yield [37,38].

Daphnia are a fundamental ecological species utilized for various biological applications including acute and chronic (eco)toxicology assessment and in fundamental research on ecology, genetics, and evolution. Due to their position in the food chain, and their filter-feeding capabilities, they are exposed to everything in their environment. Scientific data on *D. magna* related to immobilization, lethality, reproductive, behavioral, physiological and biochemical parameters used in the toxicity assessment of pharmaceuticals were presented by Tkaczyk et al. [39]. They demonstrated the role of these invertebrate model organisms for evaluating the toxicity of different therapeutic classes of pharmaceuticals. The growing need for environmental protection has led to the development of alternative biofuels. Hubai et al. [40] conducted studies related to their ecotoxicity by conducting an acute immobilization test on *Daphnia magna*. The toxic effect of CeO_2_ and ZnO nanoparticles of the same size that were stabilized by a capping agent polyvinylpyrrolidone (10 k PVP) was investigated by Briffa et al. [41]. The authors found that ZnO nanoparticles were more toxic than CeO_2_ nanoparticles and demonstrated that PVP encapsulation had no effect on toxicity. Other authors proposed a refractive index gel permeation chromatography (GPC) method for the quantification of PVP [35]. The method was applied to *Daphnia magna* exposed to PVP for 48 h. It was suggested that PVP is ingested by *Daphnia magna*, which warrants that the bioaccumulation of PVP could cause the sublethal effects observed in other studies [42,43].

The present work deals with the sol–gel synthesis and characterization of silica–poly (vinylpyrrolidone) hybrid material (SiO_2_/20PVP). Our goal was to evaluate the efficacy and toxicity of the prepared material as a potential antimicrobial agent against a broad spectrum of bacteria, including those with novel resistance mechanisms. This hybrid was tested for antibacterial efficacy alone and in combination with different conventional antibiotics against four different bacterial isolates. In addition, we started to study their redox properties and ROS-generating activity, explaining their antibacterial properties. Moreover, a toxicity test with *Daphnia magna* predicted possible maximum permissible concentration in the environment without harm for water inhabitants.

## 2. Results and Discussion

### 2.1. Phase Transformations, SEM and TEM Observations

**Figure 1:**
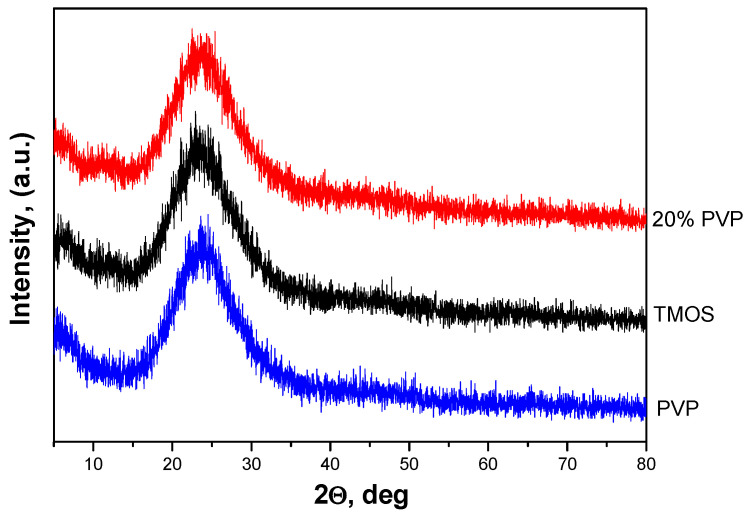
XRD patterns of SiO_2_/20PVP, TMOS, and PVP.

X-ray diffraction patterns of pure TMOS and PVP samples along with that containing 20% PVP amount are shown in Figure 1. As is seen from the figure, all gels are amorphous, which proves the hybrid material formation.

**Figure 2:**
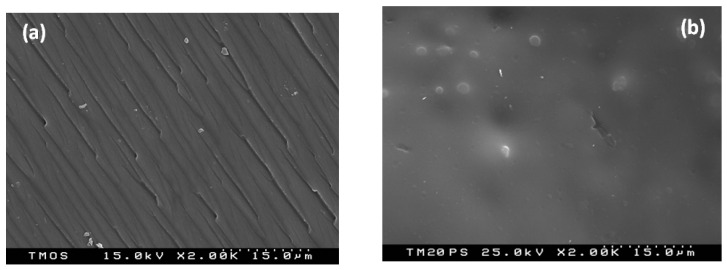
SEM micrographs of pure TMOS/SiO_2_ (**a**) and sample with 20PVP (**b**).

SEM images of the prepared gels are shown in Figure 2a,b. It is observed that the sample’s surface is homogeneous and smooth for the pure TMOS and other sample too (Figure 2a,b). This is a proof for the successful progress of the co-condensation reactions. The SEM micrographs of the hybrids containing PVP looks similar and their surface are small-grained (Figure 2b). Moreover, the surface texture of SiO_2_/20PVP (Figure 2b) shows bigger SiO_2_ grains. Obviously, the higher PVP amount leads to the increase in particle size which is about 5 µm. This was also observed by other authors [20]. Generally, it is considered that PVP belongs to the group of selective capping agents and, in a reaction, it controls the growth rates of various faces of metal oxide nanoparticles throughout the adsorption on the surfaces. In other studies, it was shown that PVP enhanced the growth rate along [100] directions [44].

**Figure 3:**
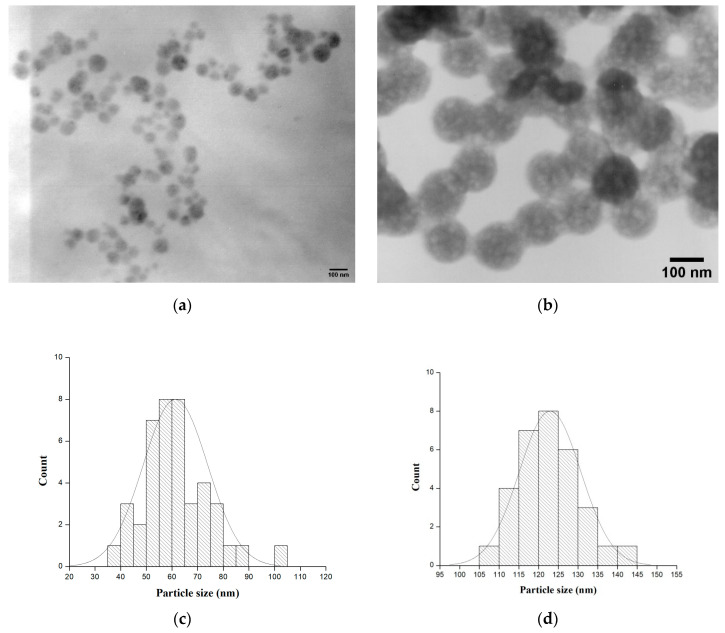
TEM images with corresponding histograms of TMOS (**a**,**c**) and SiO_2_/20PVP (**b**,**d**).

The morphology of the hybrids was also characterized by TEM. Figure 3a,b shows the TEM micrographs of the investigated samples. The images exhibit the presence of agglomeration in the material, but only in some sections, not the entire volume. As is seen from the figures, all samples—pure TMOS along with that containing 20% PVP—showed spherical particles. The particle size distribution histogram determined from TEM micrographs is shown in Figure 3c,d. It was found that the average particle size for pure TMOS was 60–65 nm while the SiO_2_/20PVP sample exhibited large amount of nanoparticles with a size of 115–125 nm. It is obvious that the increase in the PVP amount leads to an increase in the agglomeration of the samples. The obtained data through electron microscopy studies are in accordance with those obtained by other authors [45,46,47,48,49].

### 2.2. IR Structural Investigations

The IR spectroscopy is commonly used in the analysis of hybrids of organic and inorganic materials [2]. By this approach, the existence of a novel organic–inorganic network could be verified. The analysis of the absorption bands provided useful information for the molecular structure of the as-prepared materials.

**Figure 4:**
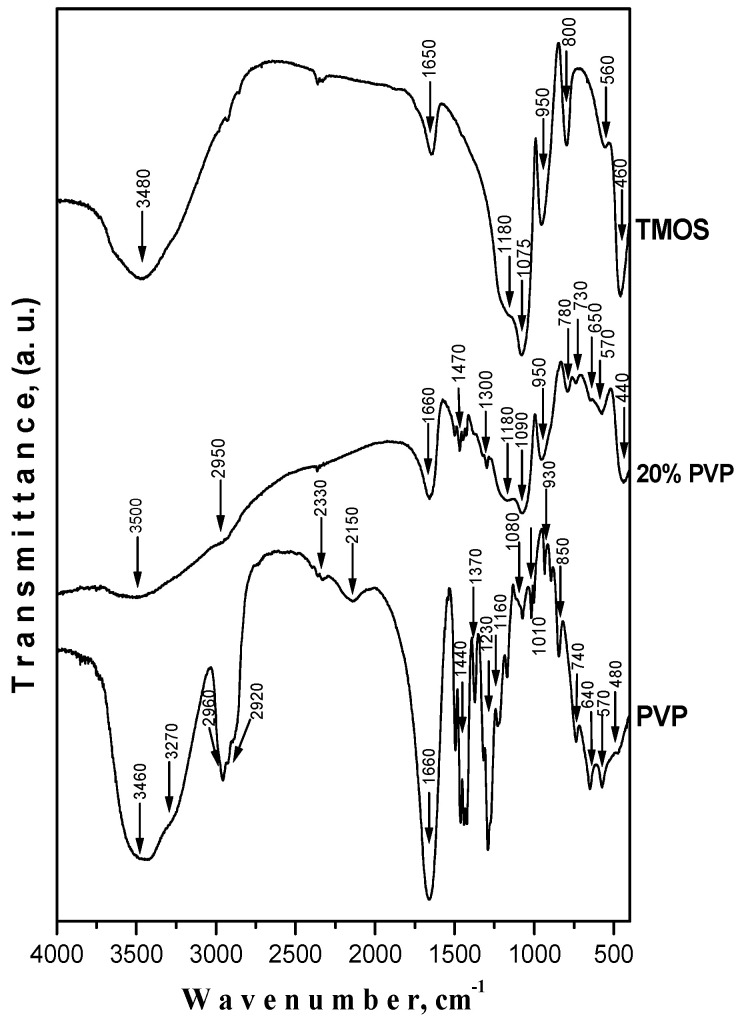
IR spectra of SiO_2_/20PVP, TMOS, and PVP.

The IR spectra of pure PVP and the sample containing 20% PVP are shown in Figure 4. In these spectra, there are several main absorption regions: 3460–2150 cm^−1^, 1700–960 cm^−1^, and 800–440 cm^−1^. It is obvious that the structures of pure TMOS, PVP, and the hybrid material are different. Typical silica bands are present in the spectra of SiO_2_/20PVP. Generally, the bands at 430, 1075, and 1180 cm^−1^ could be assigned to the bending vibrations of siloxane (Si-O-Si) bonds [50]. The characteristic band of the Si-OH is seen at about 960 cm^−1^. The absorption band at 650–640 cm^−1^ can be assigned to the Si-CH_2_ stretching [51]. Generally, the band about 1080 cm^−1^ is typical of the polymerized silica network. As is seen from the figure, this band is present and well defined in the IR spectra of the hybrid, which is an indication that the polymerized silica network was formed in the amorphous state.

In the spectra of pure PVP, a band at 1440 cm^−1^, associated with the deformations of CH_2_ groups, could be noticed. This band is shifted and transformed into a broad one centered at about 1470 cm^−1^ in the IR spectrum of the SiO_2_/20PVP hybrid. That shifting indicates a change in interchain forces in the PVP molecule. The band at 1660 cm^−1^ is present in all IR spectra, but its strongest absorption is observed in the pure PVP spectrum. This could be related to the vibrations of the C=O group which was also observed and reported by other authors [52,53]. The strong decrease in the band at 1660 cm^−1^ in the IR spectrum of the SiO_2_/20PVP sample is a proof for the presence of hydrogen bonding between PVP and SiO_2_ [54]. On the other hand, the band around 1660–1650 cm^−1^ could be also associated with the angular vibration of water molecules bonded to the inorganic framework. The wide band centered at 3500 cm^−1^ is attributed to the OH stretching frequency of the silanol groups in the inorganic framework. These findings are in good correlation with those obtained by other authors [55,56]. Overall, the spectral features show strong interactions between PVP and TMOS provoked by the contact during the sol–gel reaction.

### 2.3. ^29^Si MAS NMR Spectra

In the present study, solid-state ^29^Si NMR spectroscopy was used to investigate the structural characteristics of the silica matrix in the parent silicate and in the hybrid SiO_2_/20PVP material, while ^1^H→^13^C cross-polarization magic-angle-spinning NMR spectroscopy (^1^H→^13^C CP-MAS) was applied to get an insight into possible interactions between the PVP and the silicate framework in the hybrid SiO_2_/20PVP material [57,58,59,60].

**Figure 5:**
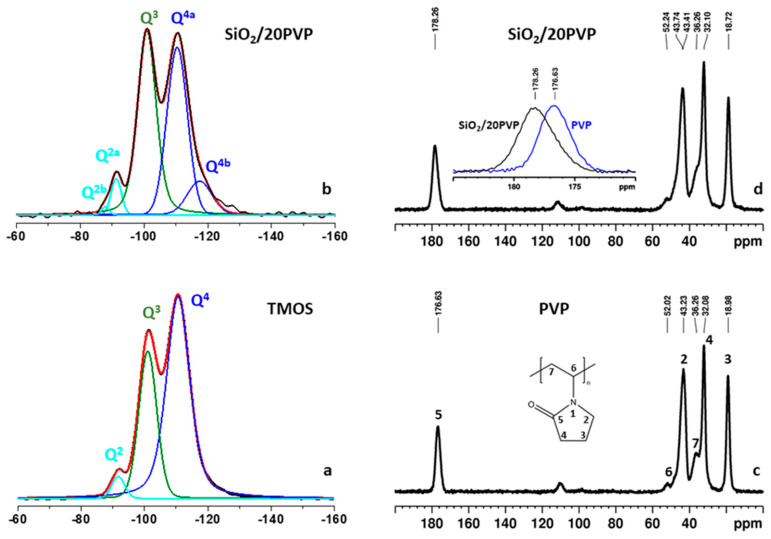
Experimental (black) and simulated (red) direct-excitation ^29^Si NMR spectra of (**a**) TMOS and (**b**) SiO_2_/20PVP. The individual contributions of the different Si environments obtained after the deconvolution are given with colored lines (Q^4^ blue, Q^3^ green, and Q^2^ light blue). (**c**) ^1^H→^13^C CP MAS spectrum of PVP. (**d**) ^1^H→^13^C CP MAS spectrum of SiO_2_/20PVP. The insert in (**d**) shows an overlay of the expanded regions of the C=O signal in the pure (blue) and hybrid (black) materials for comparison.

Chemical shifts and relative intensities of the signals in the direct-excitation (single-pulse) ^29^Si NMR spectra provide qualitative and quantitative information about the type and the distribution of the different (SiO)_n_ Si(OH)_4-n_ species (n = 1, 2, 3; e.g Q^n^ species) of the silica matrix. The single-pulse ^29^Si NMR spectrum of the parent silicate (Figure 5a) displays three partially overlapping resonances. The signal at −110.6 ppm is characteristic of the Q^4^ species [Si(0OH) structural units] that represent the main building blocks of the bulk silicate framework. The resonance at around—101 ppm is assigned to Q^3^ centers [Si(1OH) units], while the third lower intensity signal at around—91.7 ppm indicates the presence of a small amount of Q^2^ structures [Si(2OH) units]. The Q^3^ and Q^2^ units represent defect sites located on the silica surface and/or at pore edges. The relative fractions of the three types of structures were calculated by means of the deconvolution of the spectral pattern and are as follows: Q^4^:Q^3^:Q^2^ = 65:32:3. The single-pulse ^29^Si spectrum of the hybrid SiO_2_/20PVP material demonstrates a similar spectral pattern (Figure 5b); however, higher structural heterogeneity was registered in this case. The enhanced structural diversity of the hybrid material is reflected by (i) a redistribution of the intensities of the resonances for the different Si structural units, and (ii) the appearance of additional low-intensity resonances, denoted as Q^4b^ (−116 ppm) and Q^2b^ (−88 ppm), with different chemical shifts as compared to the main resonances for these type of species, denoted as Q^4a^ (−110.4 ppm) and Q^2a^ (−101 ppm). These additional resonances could be assigned to Q^4^ and Q^2^ species with distorted bond lengths and valence angles due to the incorporation of the polymer in the silica matrix. The relative fractions of the different types of Si structures in the hybrid SiO_2_/PVP material calculated by means of the deconvolution of the spectral pattern are as follows: Q^4b^:Q^4a^:Q^3^:Q^2a^:Q^2b^ = 10:40:45:4:1.

In a next step, we applied ^1^H→^13^C cross-polarization magic-angle-spinning NMR spectroscopy (^1^H→^13^C CP-MAS) to investigate the nature of the possible interactions between the PVP and the silicate in the hybrid SiO_2_/20PVP material. Figure 5c shows the ^1^H→^13^C CP-MAS spectrum of the pure PVP along with the PVP structure and the chemical shift assignment of the characteristic resonances of the respective PVP structural fragments. The spectrum of the PVP in the hybrid SiO_2_/20PVP material is given in Figure 5d. The comparison of the ^13^C chemical shifts of the two samples shows that in the spectrum of the hybrid material, the resonance of the C=O groups is shifted by 1.63 ppm toward a lower field (see the insert in Figure 5d). The signals of the other structural fragments are also shifted to a lower field; however, the chemical shift changes are either minor (up to 0.22 ppm) or remain unchanged. These observations imply that the C=O groups of the pyrrolidone polymer fragment are localized in the vicinity of the Q^3^ and Q^2^ Si centers of the silica matrix via hydrogen bonding interactions with the OH hydrogens of the silanol groups.

### 2.4. Antibacterial Properties

**Figure 6:**
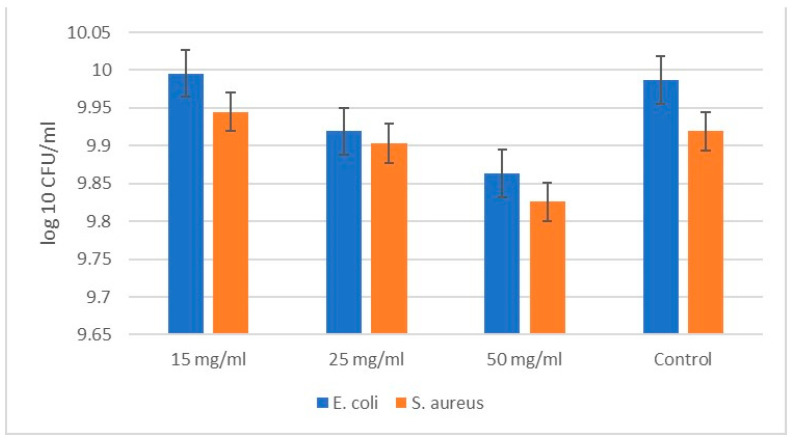
Concentration-dependent antibacterial effect of SiO_2_/20PVP on Gram-positive bacterium *Staphylococcus aureus* and Gram-negative bacterium *Escherichia coli* at 24 h.

As shown in Figure 6, the testing of the silica–poly (vinylpyrrolidone) hybrid material started at concentrations of 15 mg/mL, 20 mg/mL, and 50 mg/mL. No obvious bactericidal effect was found at these concentrations. A slight bacteriostatic effect is observed in the highest concentration (50 mg/mL) of the SiO_2_/20PVP sample. This suggested the consecutive testing of SiO_2_/20PVP in a synergistic effect with an antibiotic acting specific to Gram-negative bacteria (Ciprofloxacin) and Gram-positive bacteria (Vancomycin).

A spot-test scheme was utilized to assess the antibacterial potential of the antibiotics, the newly synthesized SiO_2_/20PVP hybrid, and their combined inhibition. The minimum bactericidal concentration (MBC) of the sample alone and in combination with antibiotics against pathogenic bacteria was examined using the broth microdilution method as described above.

**Figure 7:**
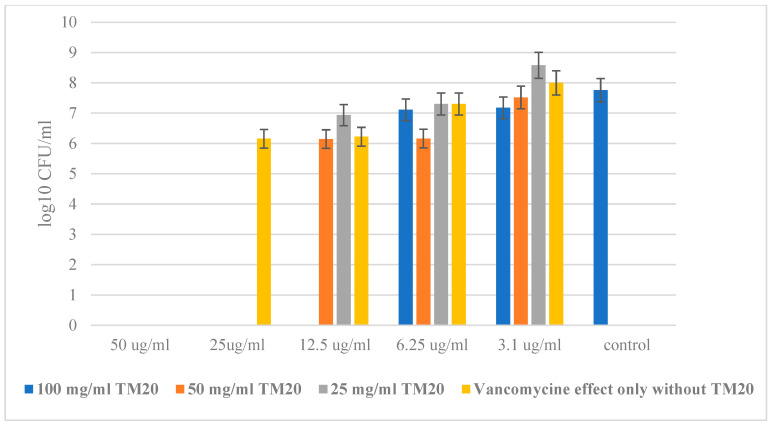
SiO_2_/20PVP in combination with Vancomycin against *Staphylococcus aureus* at 24 h.

The MBC of the single action of the antibiotic against *S. aureus* was clearly determined—50 µg/mL, while in the combination of Vancomycin with the highest concentration of SiO_2_/20PVP, the MBC already decreased to only 12.5 µg/mL for the antibiotic concentration. The results in Figure 7 show that the minimum bactericidal concentration (MBC) in combination with Vancomycin (12.5 µg/mL) of the SiO_2_/20 PVP was at a concentration of 100 mg/mL.

**Figure 8:**
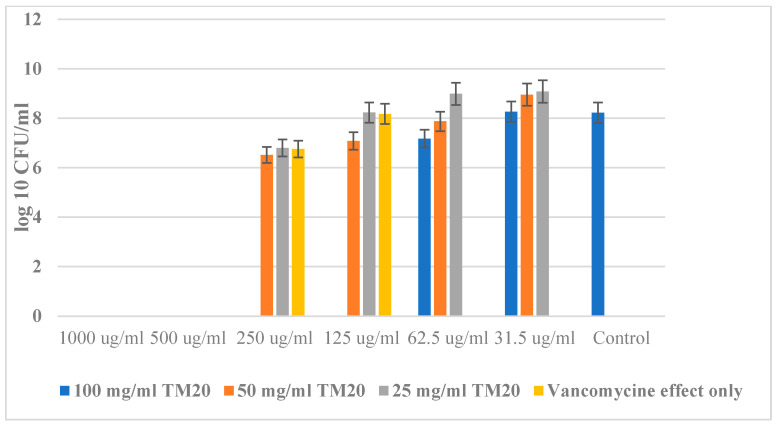
New SiO_2_/20PVP in combination with Vancomycin against *Bacillus cereus*.

The results for the Bacillus cereus are similarly presented in Figure 8. As is well known, spores of bacilli are extremely resistant to all physical and chemical agents. However, when combining 100 mg/mL of the hybrid material with the antibiotic Vancomycin at a concentration of 125 µg/mL, a bactericidal effect of two orders of magnitude was observed. It is interesting to mention that the single action of SiO_2_/20PVP at a concentration of 25 mg/mL and an antibiotic at a concentration between 30 and 60 µg/mL has a stimulating effect on this bacterium known as hormesis.

The experiments with the Gram-negative bacteria *Escherichia coli* and *Pseudomonas aeruginosa* and the antibiotic Ciprofloxacin showed similar results in the resistance of the bacteria against the antibiotic alone, and in the combination of Ciprofloxacin with the highest concentration of the hybrid. But these microorganisms are significantly more sensitive to the combination of both the antibiotic and the SiO_2_/20PVP sample. This can be explained by the difference in cell walls between Gram-positive and Gram-negative bacteria.

**Figure 9:**
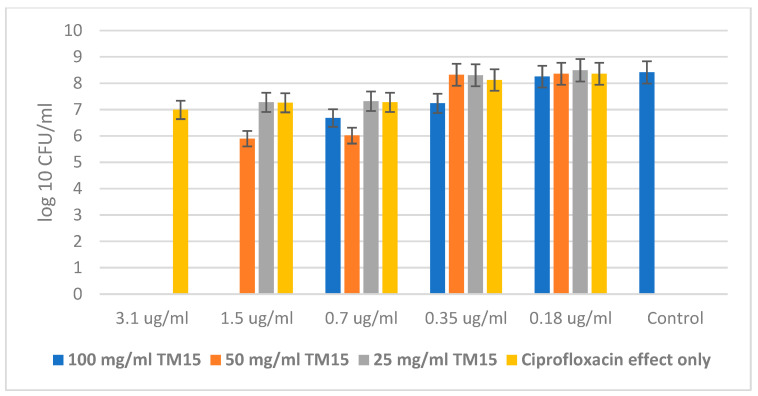
SiO_2_/20PVP in combination with Ciprofloxacin against *E. coli*.

Figure 9 shows a significantly higher MBC of Ciprofloxacin alone than in combination with 100 mg/mL of the hybrid material. The MBC of the antibiotic against *Escherichia coli* was found to be 3.5 µg/mL, while that for the combination of Ciprofloxacin and the highest concentration (100 mg/mL) of SiO_2_/20PVP was 0.7 µg/mL.

**Figure 10:**
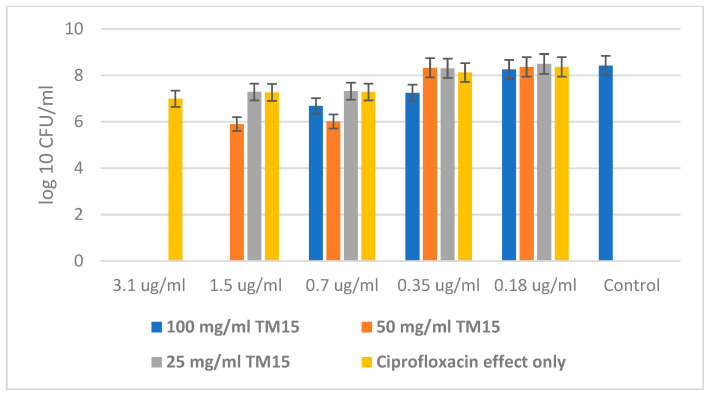
SiO_2_/20PVP in combination with Ciprofloxacin against *Pseudomonas aeruginosa*.

*Pseudomonas aeruginosa* showed higher resistance to the concentration of antibiotics. Here, the MBC of Ciprofloxacin alone was 6.25 µg/mL, while in a synergistic effect with the new hybrid material at a concentration of 100 mg/mL, the MBC decreased to 1.5 µg/mL, as is shown in Figure 10.

### 2.5. Chemiluminescence Assay

**Figure 11:**
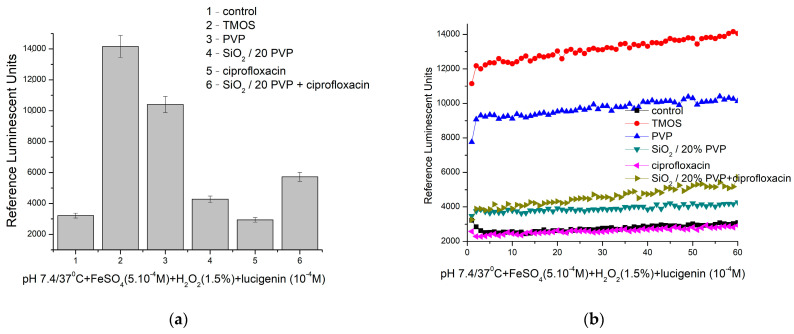
Chemiluminescence induced by ·OH and ·OOH radicals at pH 7.4 and the effect of materials: (**a**) maximum effects and (**b**) Fenton’s reaction in time (*p* ≤ 0.05).

Figure 11 presents the results about the chemiluminescence assay and the capability of the tested hybrid to generate ROS and present antibacterial properties. As a suitable, highly sensitive, and relatively inexpensive method, it allows to monitor the dynamics of free radical reactions. Thus, the prooxidant/antioxidant activity of the investigated material can be estimated [36,37].

As is seen at pH 7.4 (physiological), the kinetic curves keep a plateau shape. The Ciprofloxacin antibiotic does not affect the generation or neutralization of ROS in this model reaction. This result confirms the safe administration of the tested antibiotic in vivo and other than a free-radical generation activity mechanism. In all other cases, the recorded signal is higher than the control (more than 32,000 RLU), which is indicative of the stimulation of the generation of ·OH and ·OOH radicals. The registered effects are as follows: TMOS—340%; PVP—more than 220%; SiO_2_/20PVP—almost 33%; and SiO_2_/20PVP+ciprofloxacin—almost 78%. The as-prepared hybrid SiO_2_/20PVP shows a significant prooxidant effect, which is preserved with time, and an almost twice higher chemiluminescent signal than the control reaction. This is indicative of definite antibacterial activity. The obtained results are in agreement with the antibacterial tests conducted with all Gram-positive and Gram-negative bacteria. On the other hand, it is necessary to confirm the observed effects in additional redox model systems using the method of activated luminescence (ex vivo), at physiological pH and pH 8.5, in order to completely define the action mechanism of the tested hybrid. This will be performed in our future investigations as the obtained information will predict its effect in vivo.

### 2.6. Daphnia Magna Toxicity

**Figure 12:**
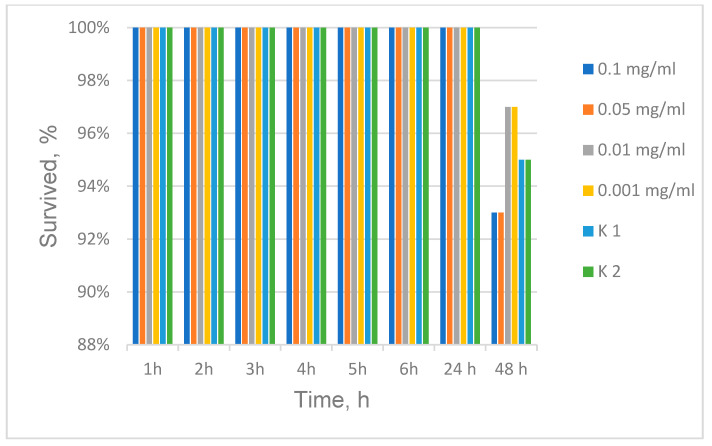
Survival of *Daphnia magna* treated with SiO_2_/20%PVP.

Aiming to estimate the toxicity of the as-prepared SiO_2_/PVP hybrid, an acute toxicity test with *Daphnia magna* was performed that allows predicting the possible maximum permissible concentration in the environment without harm for water inhabitants. The experiments were performed using four concentrations of the substance—0.1 mg/mL; 0.05 mg/mL; 0.01 mg/mL and 0.001 mg/mL with three replicates of each concentration and two controls as shown in Figure 12. Generally, *D. magna* are extremely sensitive organisms in the aquatic environment, which also allows conclusions to be drawn from the obtained results about the influence of the as-prepared hybrid on other aquatic organisms. The results of the survival of *Daphnia* were 97% at a concentration of 0.01 and 0.001 mg/mL and 93% at a concentration of 0.1 and 0.05 mg/mL of the substance. Since 95% *D. magna* survival was observed in the controls, it can be speculated that low concentrations below 0.01 mg/mL have a slight stimulatory effect, while the higher concentration of 0.05 mg/mL has a weak inhibitory effect. Therefore, at these concentrations, the substance used can be discharged into surface waters without harm to organisms.

### 2.7. Analysis of the Results

Bearing in mind the obtained results, it has to be noted that one experimental fact still provokes scientific interest. Irrespective of the absence of antibacterial activity of the sol–gel-prepared pure SiO_2_/20PVP hybrid, in a synergistic effect with antibiotics, it acts specific to both Gram-negative and Gram-positive bacteria. It is evident that the functionalization of the silica hybrid with small molecules such as vancomycin and ciprofloxacin provokes the antibacterial activity of the hybrid. Despite the differences in the cell wall of the tested Gram-positive (*Staphylococcus aureus* and *Bacillus cereus*) and Gram-negative (*Escherichia coli* and *Pseudomonas aeruginosa*) bacteria, it was established that the combination of both the antibiotic and the SiO_2_/20PVP sample led to a significant increase in the antibacterial properties. According to Bernardos et al. [61], this experimental result could be ascribed to the fact that the antibiotic reacts with the outer surface of the hybrid, forming a hydrogen bonding interaction with the terminal functional groups on the cell membrane. Supporting this suggestion are the performed IR and NMR analyses which prove the presence of hydrogen bonding interactions between the silica matrix and the OH hydrogens of the silanol groups (Figure 4 and Figure 5). Moreover, the registered prooxidant activity coincides well with the suggested antibacterial properties of the tested substance and its combination with antibiotics. On the other hand, it has already been stated that the size, shape, pore diameter, surface properties, composition, and concentration features can all contribute to these properties of the synthesized material [62,63,64]. For example, some authors established that sol–gel-derived PVP powders composed of spherical particles exhibited higher antibacterial properties as compared to ellipsoid ones [65]. This experimental fact corresponds well to the results obtained by us. Moreover, the investigated hybrid exhibited good killing efficiency at the 24th hour.

One complementary investigation concerning the *D. magna* toxicity test highlights this paper’s originality. It was observed that the effect of the hybrid material on Crustacea *D. magna* is estimated to be harmless at a concentration of 0.1 mg/mL. This could be related with the fact that the silica-based compounds are hydrolytically unstable and they progressively transform into harmless silicic acid or polysilicic acid that the body can safely absorb or excrete [61]. It is evident that, at these concentrations, the sol–gel-derived substance can be discharged into surface waters without harm to aquatic organisms.

Thus, it could be generalized that the present study, which is devoted to obtaining a SiO_2_-containing hybrid material and evaluating its antibacterial properties, represents an attempt to elucidate the composition–structure–property relationship. Based on the above discussed results, it could be suggested that the as-prepared material can be used in further research related to other possible applications of SiO_2_/20PVP for environmental protection.

## 3. Experimental Procedure

### 3.1. Materials

The gels were prepared using a combination of the alkoxid—tetramethyl orthosilane (TMOS)—Sigma Aldrich Chemical (Burlington, MA, USA) and poly(vynyl pyrrolidon) K25-(PVP), Fluka Chemie AG (Buchs, Switzerland). The preparation procedure is described in details below.

### 3.2. Preparation of Silica–Polyvinylpyrrolidone (SiO_2_-PVP) Hybrids

**Figure 13:**
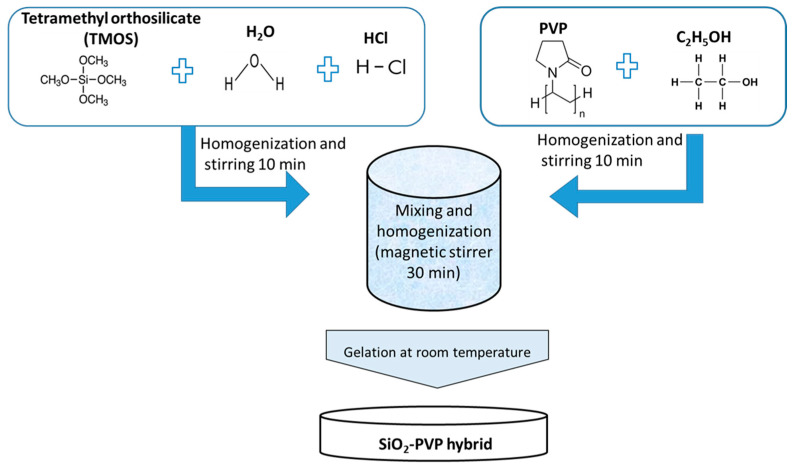
Scheme for the synthesis of SiO_2_/PVP hybrids.

The precursor tetramethyl orthosilicate {TMOS, Si(OCH_3_)_4_}, distilled H_2_O, and 0.1 M HCl in the molar ratio 4:1:1 were mixed and stirred together. Aiming to obtain a hybrid material with improved properties, the added amount of PVP was 20wt %. It was dissolved in ethanol and added dropwise to the stirred solution. Stirring continued until the mixture became clear. The gelation occurred at room temperature and it took about 20 h. All steps of the hybrid preparation are shown in Figure 13. The investigated samples were denoted as TMOS, PVP, and SiO_2_/20PVP.

### 3.3. Methods of Characterization

Powder X-ray diffraction data were obtained on a Philips PW1730/10 diffractometer using Ni-filtered CuKα radiation (Philips Corporation, Almelo, the Netherlands). The scanning rate for crystallinity was 1.2º 2θ/min. SEM images were obtained on a Hitachi S-4100 (Hitachi Ltd., Tokyo, Japan) microscope at an accelerating voltage of 25.0 kV. TEM images were taken on a Hitachi H-600A (Tokyo, Japan). A powdered hybrid sample was suspended in a mixture of ethanol and polysorbate 80 (1:1 *v/v*) using an ultrasonic generator (with an intensity of 250 W) for 3 min. One or two drops of the resulting dispersion were dropped on a 300-mesh copper grid (Tedpella, Inc., Redding, CA, USA) coated with carbon film and left to evaporate. FTIR spectra were registered using the KBr pellet technique on an FTIR spectrophotometer—Nicolet 6700 FT (Matson, UK). ^29^Si MAS NMR spectra were recorded on a Bruker Avance HD III 600 NMR spectrometer (Billerica, MA, USA) operating at 599.98 MHz proton frequency (150.84 MHz for ^13^C, 119.18 MHz for ^29^Si), using 4 mm solid state i-CP/MAS dual ^1^H/^31^P-^15^N probehead (Billerica, MA, USA). The samples were loaded in 4 mm zirconia rotors and spun at a magic-angle-spinning (MAS) rate of 10 kHz for both the ^13^C and ^29^Si measurements. ^1^H→^13^C cross-polarization MAS (CP MAS) spectra were acquired with the following experimental parameters: 8 K time domain data points, a spectrum width of 50 kHz, an ^1^H excitation pulse of 3.6 µs, a contact time of 2 ms, 2048 scans, and a recycle delay of 5 s. The ^1^H SPINAL-64 decoupling scheme was used during the acquisition of the CP experiments. The spectra were processed with an exponential window function (line broadening factor 10) and zero-filled to 32 K data points. The quantitative direct-excitation ^29^Si NMR spectra were recorded with one-pulse sequence, a 90° pulse length of 4.5 µs, 3 K time domain data points, a spectrum width of 70 kHz, 1024 scans, and a relaxation delay of 60 s. Exponential window function was applied (line broadening factor 10) prior to Fourier transformation.

### 3.4. Antimicrobial Activity Testing

The tested microbial strains, Gram-negative bacteria *Escherichia coli* ATCC 25922 and *Pseudomonas aeruginosa* ATCC 27853, and Gram-positive bacteria *Staphylococcus aureus* ATCC 25923 and *Bacillus cereus* NBIMCC 1090, were provided by the National Bank of Microorganisms and Cell Cultures (NBIMCC), Bulgaria, and cultured in the nutrient medium (Conda, Spain). The pure culture of every strain was prepared as a bacterial suspension in exponential phase with OD 0.5 McFarland.

The SiO_2_/20PVP sample was tested for antibacterial efficacy alone and in combination with conventional antibiotics in liquid medium too. Antibiotics were selected from five groups with a different mode of action, including vancomycin (Glycopeptide) and ciprofloxacin (Quinolone). Vancomycin is a tricyclic glycopeptide antibiotic used for the treatment of Gram-positive bacterial infections. It belongs to the cell-wall-synthesis inhibitor class of antimicrobial drugs. Almost all quinolone antibiotics used are fluoroquinolones, which contain a fluorine atom in their chemical structure and are effective against both Gram-negative and Gram-positive bacteria [66].

### 3.5. Methodology for the Evaluation of the Minimum Inhibitory and Minimum Bactericidal Concentration (MBC) and Antibacterial Mode of Inhibition of SiO_2_/20PVP

The MBC of the SiO_2_/20PVP hybrid combined with antibiotics was determined using the broth microdilution method and spot-test on agar. A standard suspension of bacterial pathogens (100 μL/0.5 MFU) was added to micro dilution wells containing 50 μL of the corresponding concentration of the investigated sample (25 mg/mL, 50 mg/mL, and 100 mg/mL) and 50 μL of different concentrations of the antibiotic for each well to obtain a final volume of 200 µL in 11 variants. The final (12th) column was left for control (100 μL bacterial suspension and 100 μL autoclaved physiological saline solutions). After 24 h incubation at 36 ± 1 °C, the wells were examined for growth. The lowest concentration that showed no growth was assessed as a minimum bactericidal concentration (MBC). It can be determined from broth tests by sub-culturing to agar plates that do not contain the tested agent [30]. Experiments were performed with three independent runs of three replicates per run.

### 3.6. Chemiluminescence Assay

#### 3.6.1. Materials

Purchased with high purity: iron sulphate (p. a.) (Merck, Darmstadt, Germany), hydrogen peroxide (30%) (Merck, Darmstadt, Germany), lucigenin (bis-N-methylacridinium nitrate) (p. a.) (Sigma-Aldrich, St. Louis, MI, USA and Burlington, MA, USA), dimethyl sulfoxide (p. a.) (DMSO, Sigma-Aldrich, St. Louis, MI, USA), and buffer pH 7.4 (Sigma-Aldrich, St. Louis, MI, USA and Burlington, MA, USA). All chemicals were used as purchased.

#### 3.6.2. Method

The redox activity of the newly synthesized material was tested in Fenton’s model chemical reaction system, generating free radicals and ROS, at pH 7.4 (physiological), by means of the activated chemiluminescence method [67]. The chemical probe that we applied for signal amplification was lucigenin. Thus, reliable, comparable differences were achieved.

Fenton’s system generates ^.^OOH and ^.^OH radicals. The control samples do not contain the tested material (active concentration 100 mg/mL). The reactions are monitored for 3 min, every 3 s, at 37 °C, and measured in triplicate. All materials are sonicated for 20 min prior to testing to ensure good dispersion.

Fenton’s system:

Fe^2+^ + H_2_O_2_ → Fe^3+^ + ^·^OH + ^−^OH (1)

Fe^3+^ + H_2_O_2_ → Fe^2+^ + ^·^OOH + H^+^ (2)

In general, the higher the signal, the more ROS are formed and a prooxidant effect is described. On the contrary, if the signal is below the control level, antioxidant activity can be described.

#### 3.6.3. Statistics

All experiments were performed using LUMIstar Omega (BMG Labtech GmbH, Ortenberg, Germany, 2020) in triple reproducible measurements; statistical analysis was performed using OriginPro 8 and Microsoft Office Excel 2010.

### 3.7. Daphnia Magna

A toxicity test was conducted according to the acute lethality toxicity protocol OECD (2004) and the guidelines for the testing of chemicals’ acute immobilization test (2012), Test No: 211 [68]. The experiments used 4 concentrations of the hybrid SiO_2_/20PVP nanoparticles (0.1 mg/mL; 0.05 mg/mL; 0.01 mg/mL; and 0.001 mg/mL) with 3 replicates of each concentration and 2 controls.

## 4. Conclusions

Hybrid nanocomposites containing TMOS and PVP were synthesized via the sol–gel method at room temperature. The obtained products were characterized using several methods. They established homogeneous and amorphous samples which showed useful structural properties. The IR and ^29^Si MAS NMR spectra proved the formation of a polymerized silica network as well as the hydrogen bonding interactions between the silica matrix and the OH hydrogens of the silanol groups. The TEM showed spherical particle formation along with increased agglomeration tendency.

The minimum bactericidal concentration (MBC) in the synergistic effect of the hybrid material (SiO_2_/20PVP) with the corresponding antibiotic was three to five times lower than in the case of the antibiotic action alone. These findings suggest that the synthesized SiO_2_/20PVP hybrid may be a new, effective, and broad-spectrum antibacterial agent, even at lower doses than those currently used in clinical trials to treat bacterial infections. The observed prooxidant activity confirmed and explained the registered antibacterial properties of the tested substance and its combination with antibiotics, especially ciprofloxacin. The low concentrations below 0.01 mg/mL had a slight stimulatory effect on *Daphnia magna*, while the higher concentration of 0.05 mg/mL had a weak inhibitory effect. Therefore, at these concentrations, the substance can be discharged into surface waters without harm to water organisms.

## Data Availability

Data are contained within the article.
